# How Far Can We Go with Laparoscopic Liver Resection for Hepatocellular Carcinoma? Laparoscopic Sectionectomy of the Liver Combined with the Resection of the Major Hepatic Vein Main Trunk

**DOI:** 10.1155/2015/960752

**Published:** 2015-08-27

**Authors:** Zenichi Morise, Norihiko Kawabe, Hirokazu Tomishige, Hidetoshi Nagata, Jin Kawase, Satoshi Arakawa, Masashi Isetani

**Affiliations:** Department of Surgery, Fujita Health University School of Medicine Banbuntane Houtokukai Hospital, 3-6-10 Otobashi Nakagawa-ku, Nagoya, Aichi 454-8509, Japan

## Abstract

Although the reports of laparoscopic major liver resection are increasing, hepatocellular carcinomas (HCCs) close to the liver hilum and/or major hepatic veins are still considered contraindications. There is virtually no report of laparoscopic liver resection (LLR) for HCC which involves the main trunk of major hepatic veins. We present our method for the procedure. We experienced 6 cases: 3 right anterior, 2 left medial, and 1 right posterior extended sectionectomies with major hepatic vein resection; tumor sizes are within 40–75 (median: 60) mm. The operating time, intraoperative blood loss, and postoperative hospital stay are within 341–603 (median: 434) min, 100–750 (300) ml, and 8–44 (18) days. There was no mortality and 1 patient developed postoperative pleural effusion. For these procedures, we propose that the steps listed below are useful, taking advantages of the laparoscopy-specific view. (1) The Glissonian pedicle of the section is encircled and clamped. (2) Liver transection on the ischemic line is performed in the caudal to cranial direction. (3) During transection, the clamped Glissonian pedicle and the peripheral part of hepatic vein are divided. (4) The root of hepatic vein is divided in the good view from caudal and dorsal direction.

## 1. Introduction

Tumor location, besides the tumor stage and liver function, had been among the operative indications for laparoscopic liver resection (LLR) for years since the first introduction of the procedure [1]. The most favorable locations for LLR had been anterolateral liver segments (segments 2–6) [2, 3]. The limitations of the indications associated with LLR have gradually diminished with the recent accumulation of experiences and technological advances for devices. Although the reports are still few in number, LLR has been shown to be feasible and safe also for lesions in posterosuperior segments (segments 1, 7, and 8) [4–7]. However, the centrally located tumors close to the liver hilum and/or major hepatic veins are still considered contraindications, even though the reports of laparoscopic major hepatectomy are increasing. Parenchymal transection near these major vessels has high risk for injury which may be difficult to handle laparoscopically [3]. There are very few reports of LLR for those lesions [8]. On the other hand, liver tumors, especially hepatocellular carcinoma (HCC), often involve major hepatic veins. The findings of convoluted main trunk of the veins in tumor capsules and macroscopic tumor thrombus to the veins of HCC are the indicators of combined major hepatic vein resection. Although hemihepatectomy or central bisectionectomy would be applied to such cases with well-preserved liver function, extended sectionectomy of the liver combined with the major hepatic vein resection is often applied to the cases with deteriorated liver function like HCC with chronic liver disease (CLD). This procedure could achieve R0-1 resection for HCC with thick nontumorous fibrous capsule; however, it is complicated and difficult even in open liver resection [9].

To our knowledge, there is virtually no technical report of LLR procedures for the tumors which involve major hepatic veins. We herein present our method of handling pure laparoscopic extended sectionectomy of the liver combined with the major hepatic vein resection at its root. We believe that this is the first technical report for this procedure ever.

## 2. Methods

We experienced 98 pure LLR, including 36 anatomical resections until December 2014. Among them, there are 6 cases of pure laparoscopic extended sectionectomy of the liver combined with the resection of the major hepatic vein main trunk for HCC patients (Table 1; case numbers in the table are listed in chronological order in our experience). Three right anterior sectionectomies combined with middle hepatic vein resection, 3 left medial sectionectomies combined with middle hepatic vein resection, and 1 right posterior sectionectomy combined with right hepatic vein resection are included in this series. All patients underwent LLR for hepatocellular carcinoma with chronically injured liver. The sizes of the tumors are 40–75 (median: 60) mm. One patient underwent extended right anterior sectionectomy combined with middle hepatic vein resection due to macroscopic tumor thrombus up to the first bifurcation of the vein main trunk. The others underwent extended sectionectomy combined with major hepatic vein resection due to the findings of convoluted main trunk of the vein in tumor capsules.

### 2.1. Surgical Procedures

**Figure 1:**
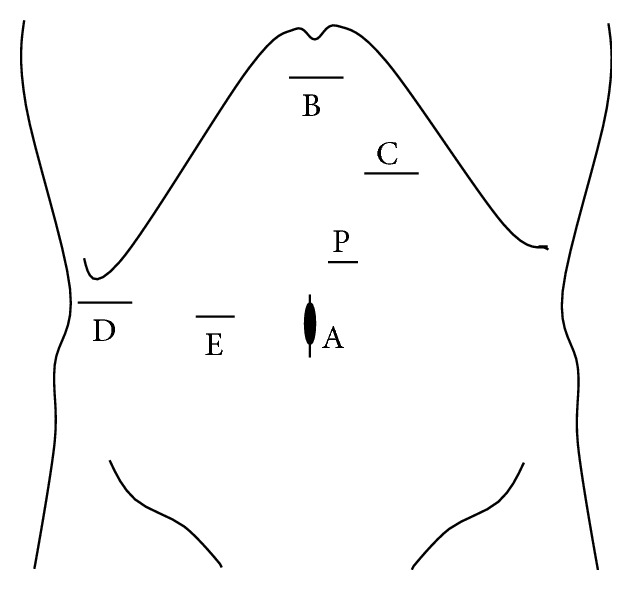
Port arrangement of extended left medial sectionectomy. A: 1st port which is inserted under minilaparotomy and mainly used as a camera port. B–D: 12 mm ports. E: 5 mm port. P: A Nelaton catheter with vessel tape inside Pringle maneuver was directly inserted through this 5 mm port size hole. The operator mainly used B, C for left-side and D, E for right-side parenchymal transection. The assistant mainly used the other ports for suction and retraction of the liver.

**Figure 2 fig2:**
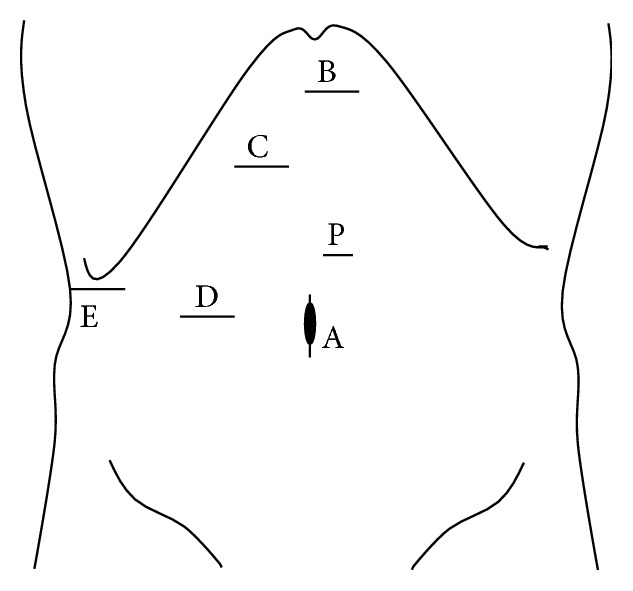
Port arrangement of extended right anterior sectionectomy. A: 1st port which is inserted under minilaparotomy and mainly used as a camera port. B–E: 12 mm ports (the port for E was converted from 5 mm to 12 mm during the operation). P: A Nelaton catheter with vessel tape inside Pringle maneuver was directly inserted through this 5 mm port size hole. The operator mainly used B, C for left-side and D, E for right-side parenchymal transection. The assistant mainly used the other ports for suction and retraction of the liver.

**Figure 3:**
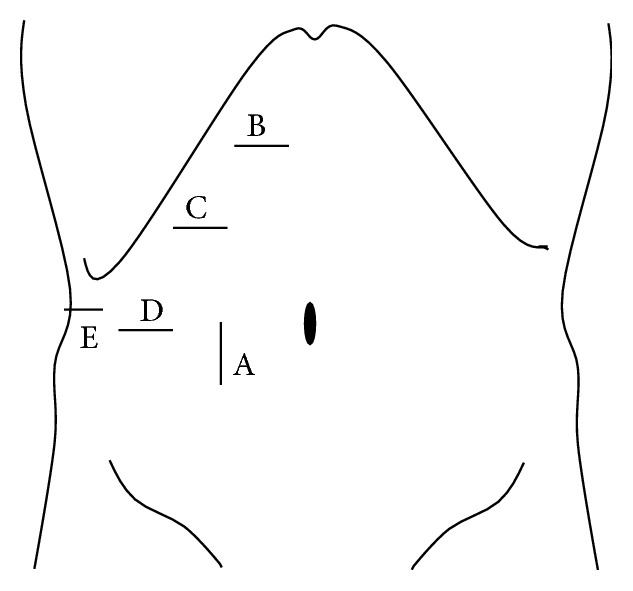
Port arrangement of extended right posterior sectionectomy. A: 1st port which is inserted under minilaparotomy and mainly used as a camera port. B–D: 12 mm ports. D: 5 mm port. The operator mainly used B–D for parenchymal transection and the assistant mainly used E and D (or C) for suction and retraction of the liver.

Patients were placed primarily in supine position for left medial sectionectomy, left hemilateral position for right anterior sectionectomy, and left lateral position for right posterior sectionectomy. Typical placements of the ports for each procedure are described in Figures 1 [Fig fig2]–3.

Summary of the common surgical procedure is listed below:

1. The Glissonian pedicle of the section is enciecled and clamped under the laparoscopic magnified view (not divided; however, in left medial sectionectomy, the Glissonian pedicle is divided at this time).
2. Liver parenchymal transection is performed on the ischemic line, as straight as possible, in the caudal to cranial direction (part of the transaction line protrudes the ischemic line due to combined resection of hepatic vein).
3. During transection, the clamped Glissonian pedicle and the peripheral part of hepatic vein are divided with lineal stapler at the time of the transection line reaching the level of the structures on both sides.
4. After the completion of parenchymal transaction, good view and approach to the root of the hepatic vein are obtained from caudal and dorsal direction. The vein is divided with lineal stapler safely under the laparoscopic magnified view and the resection is completed.

**Figure 4:**
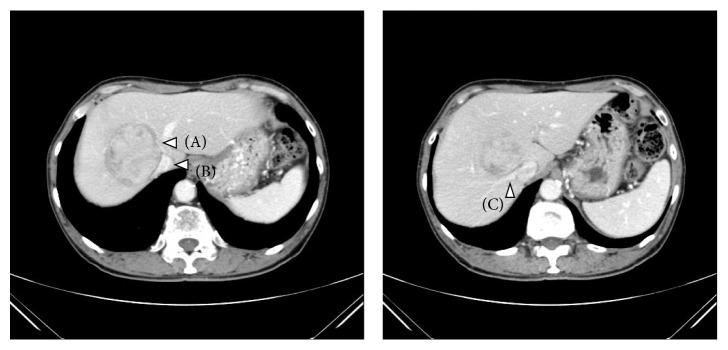
Preoperative CT findings of case number 3 (right anterior sectionectomies combined with middle hepatic vein resection). 58-year-old man with type-C chronic hepatitis developed 7 cm HCC in segment 8–1. The tumor compressed the inferior vena cava (B) and the right hepatic vein (C) widely and also there was an involvement of the middle hepatic vein (A). This patient underwent extended right anterior sectionectomy combined with the middle hepatic vein resection at its root. This case is one of the most complicated cases for pure laparoscopic liver resection due to the long dissection of the inferior vena cava to the right hepatic vein and the resection of the middle hepatic vein at its root.

**Figure 5 fig5:**
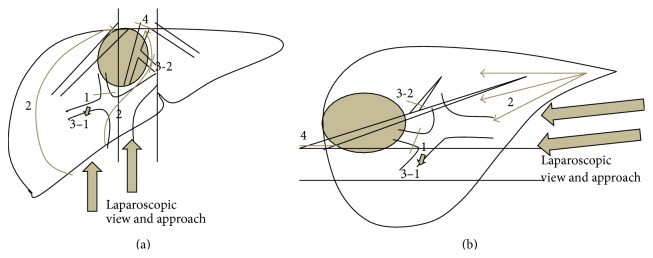
Schema of the steps for case 3 operation (right anterior sectionectomies combined with middle hepatic vein resection). (a) Front view and (b) side view. Step  1 is as follows: the anterior branch of Glissonian pedicle is encircled and clamped under the laparoscopic magnified view. Step  2 is as follows: transection of the liver parenchyma on the ischemic line, as straight as possible, in the caudal to cranial direction is performed. (Part of the left transaction line protrudes the ischemic line due to combined resection of middle hepatic vein.) Step  3-1 is as follows: the clamped anterior Glissonian pedicle is divided with lineal stapler at the time of the transection line reaching the level of the structures on both sides, during liver transaction. Step  3-2 is as follows: the peripheral part of middle hepatic vein is divided with lineal stapler at the time of the transection line reaching the level of the structures on both sides, during liver transaction. Step 4 is as follows: after the completion of parenchymal transaction, good view and approach to the root of middle hepatic vein are obtained from caudal and dorsal direction. The vein is divided with lineal stapler under the laparoscopic magnified view and the resection is completed.

**Figure 6 fig6:**
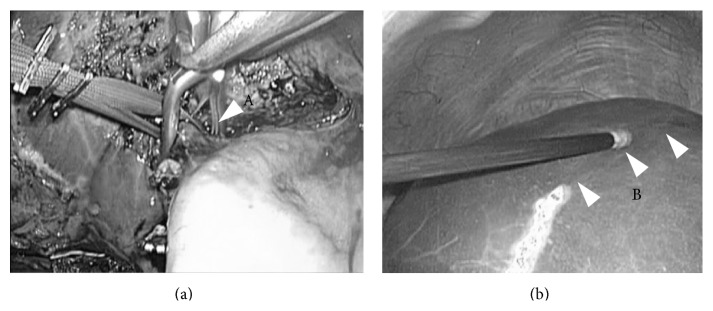
Encircling and clamping of the Glissonian pedicle of the section (intraoperative findings of case 3). At the first step after cholecystectomy, the right anterior branch of Glissonian pedicle was encircled, taped, and clamped with detachable Bulldog Clamp (left). Liver transection was started on the ischemic line with the clamp (not transection) of the pedicle (right).

**Figure 7 fig7:**
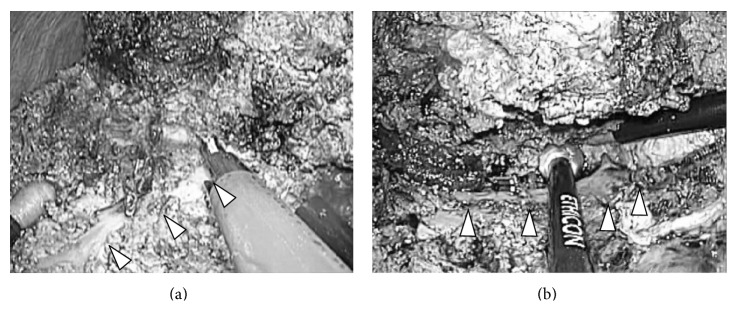
Liver parenchymal transection (intraoperative fidings of case 3). Liver parenchymal transection was performed as straight as possible in the caudal to cranial direction. The surface of the right hepatic vein (arrow head) was simultaneously dissected pursuing its root in the right transection plane of this case.

**Figure 8 fig8:**
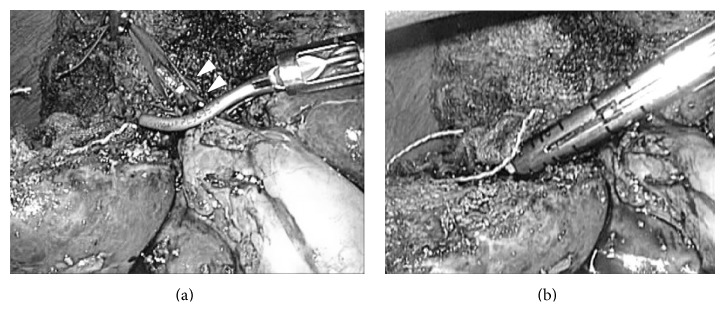
Division of the Glissonian pedicle (intraoperative findings of case 3). During transection, the clamped Glissonian pedicle (arrow head) was divided with lineal stapler (right) at the time of the transection line reaching the level of the structures on both sides.

**Figure 9 fig9:**
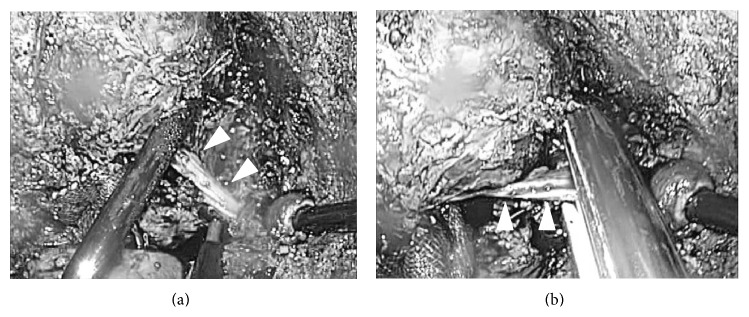
Division of the distal part of hepatic vein (intraoperative findings of case 3). During transection, the distal part of hepatic vein (arrow head) was well dissected and divided with lineal stapler (right) at the time of the transection line reaching the level of the structures on both sides.

**Figure 10 fig10:**
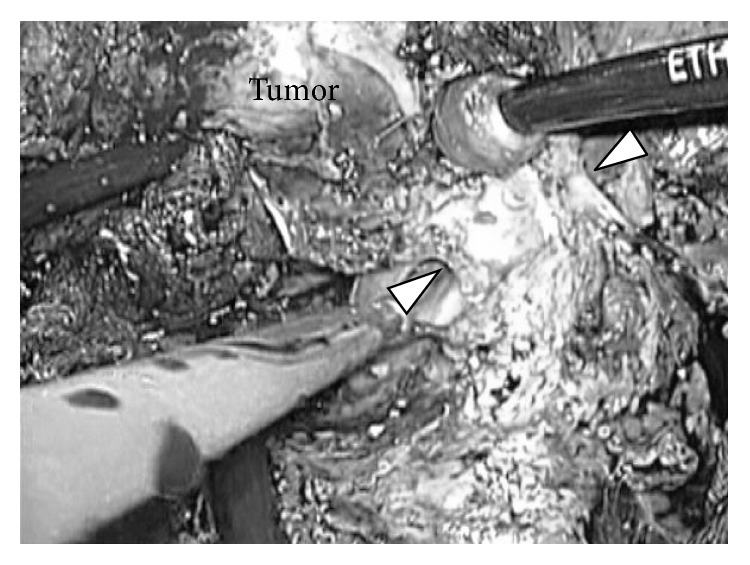
Last step of resection. View for the root of hepatic vein (intraoperative findings of case 3). After completion of liver parenchymal transaction, the root of hepatic vein (arrow head) was clearly observed in the laparoscopic magnified view from caudal and dorsal direction. The root of hepatic vein was divided safely with lineal stapler and specimen was removed thereafter.

**Figure 11:**
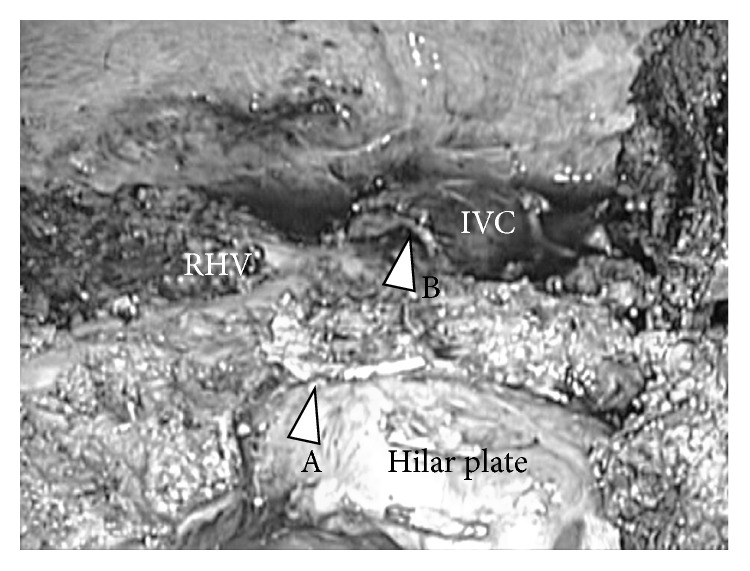
Intraoperative findings after completion of the resection, case 3. After completion of liver resection, IVC, right hepatic vein (RHV), hilar plate, the stump of right anterior Glissonian pedicle (arrow head A), and the stump of middle hepatic vein (arrow head B) were clearly shown in the operative field.

**Figure 12:**
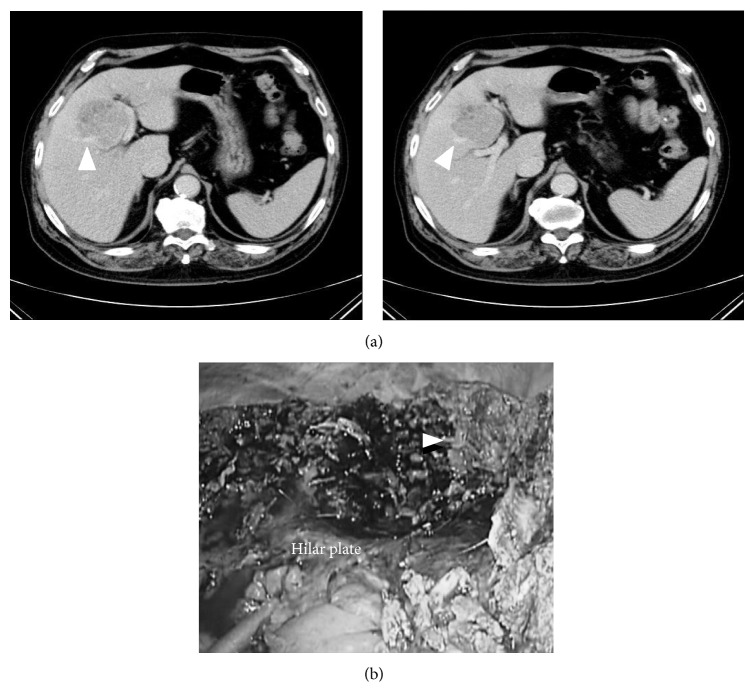
Preoperative CT findings (a) and intraoperative findings after completion of the resection (b), case 2 (left medial sectionectomies combined with middle hepatic vein resection). 70-year-old man with type-C chronic hepatitis developed 7.5 cm HCC in segment 4–8. (a) The tumor compressed the Glissonian pedicle widely, from hilar plate to umbilical plate and to anterior branch, and also there was an involvement of the middle hepatic vein (arrow head). This patient underwent extended left medial sectionectomy combined with the middle hepatic vein resection. (b) After completion of liver resection, the Glissonian pedicle, from hilar plate to umbilical plate and to anterior branch, was exposed and the stump of middle hepatic vein (arrow head) was clearly shown in the operative field.

**Figure 13:**
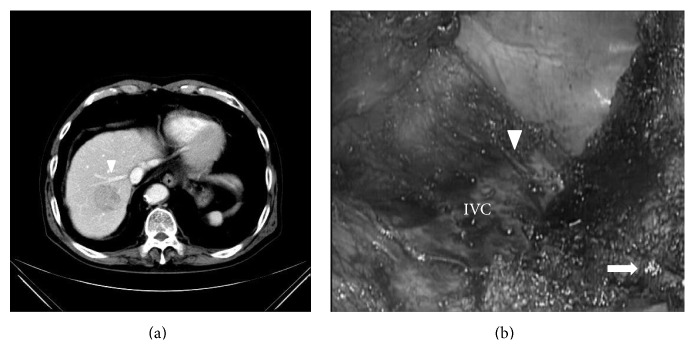
Preoperative CT findings (a) and intraoperative findings after completion of the resection (b), case 4 (right posterior sectionectomies combined with right hepatic vein resection). 70-year-old man with alcoholic chronic hepatitis developed 4 cm HCC in segment 7. (a) The tumor compressed the right hepatic vein (arrow head). This patient underwent extended right posterior sectionectomy combined with the right hepatic vein resection at its root. (b) After completion of liver resection, IVC, the stumps of right posterior Glissonian pedicle (arrow), and right hepatic vein (arrow head) were clearly shown in the operative field.

Examples of detailed steps of the procedures are described in Figures 4 [Fig fig5] [Fig fig6] [Fig fig7] [Fig fig8] [Fig fig9] [Fig fig10]–11, schema of the procedure, and pre- and intraoperative pictures from case 3 (right anterior sectionectomies combined with middle hepatic vein resection). Also, pre- and intraoperative pictures from cases 2 (left medial sectionectomies combined with middle hepatic vein resection) and 4 (right posterior sectionectomies combined with right hepatic vein resection) are listed in Figures 12 and 13.

In these procedures, the mobilization of the right liver was not usually performed. The roots of hepatic veins were minimally dissected only to confirm the end point of transection plane at the first step. Even in extended right posterior sectionectomy, the dissection of the retroperitoneal attachments was performed at the final step of the surgery after completion of the parenchymal transection. We had previously reported and described a caudal approach to posterior sectionectomy with parenchymal transection prior to mobilization of the liver [10]. In this procedure, the patient was placed in left lateral position, in which the cutting plane for posterior sectionectomy turns to be vertical from horizontal. The posterior section was not mobilized and fixed to the retroperitoneum during transection. Since the remnant liver sinks down and the resected liver was fixed to the retroperitoneum, the cutting surface was well opened and the exposure of hepatic vein for the control of bleeding was facilitated.

Pringle maneuver was applied to all cases. CUSA, BiClamp bipolar forceps, and irrigation monopolar electric cautery with soft-mode coagulation, beside Pringle maneuver and the control of pneumoperitoneal pressure, were used mainly through the operator's right or left hand port (Figures 1–3) for hemostasis and dissection without bleeding during the procedures.

We examined the tumor size and short-term outcomes (operation time, amounts of intraoperative bleeding, mortality, morbidity, and duration of postoperative hospital stay) of the cases, to compare them with the outcomes of the other 29 patients with conventional pure laparoscopic anatomical resection (13 HCC, 11 metastatic tumors, 3 gall bladder carcinoma, and 2 benign tumor; tumor size: 12–145 (23) mm), and summarized our steps of procedures.

#### 2.1.1. Statistical Analysis

Groups were compared using Student's *t*-test, Fisher's exact test, and Chi-squared test on SPSS version 11 statistical software (SPSS Inc., Chicago, IL, USA), with *P* < 0.05 indicating significance.

## 3. Results

The operation times of the cases who underwent pure laparoscopic extended sectionectomy combined with major hepatic vein resection are during 341–603 (median: 434) minutes. Intraoperative bleeding amounts are during 100–750 (median: 300) mL. Postoperative hospital stays are during 8–44 (median: 18) days. (Table 1) There was no mortality and 1 patient developed postoperative pleural effusion to be treated with drain insertion. Another patient had 44 days postoperative hospital stay with his unstable warfarin control after surgery, which had been given for his cardiac pacemaker before surgery, but without any other specific complications.

In the comparison with the results of the other 29 patients with conventional pure laparoscopic anatomical resections (operation time: 217–848 (401) minutes; bleeding amount: not countable, 3569 (181) mL; no mortality; complication: 5 out of 29 patients; postoperative hospital stay: 8–52 (15) days), these cases with pure laparoscopic extended sectionectomy have tendency with larger tumor size, longer operation time, and larger amount of intraoperative bleeding. However, there is no statistically significant difference. Also, the rate of morbidity (1/7 and 5/29 for extended sectionectomy and other anatomical resections) is similar and both groups have no mortality (Table 2).

## 4. Discussion

Right or left hemihepatectomy or central bisectionectomy, which is, of necessity, combined with the major hepatic vein resection, had been shown to be feasible and safe in studies, recently also in the donor hepatectomies [11, 12]. In the cases with the findings of convoluted main trunk of the veins in tumor capsules and macroscopic tumor thrombus to the veins, hemihepatectomy or central bisectionectomy is usually applied for the resection. However, the rate of mortality and morbidity after major hepatectomy is not negligible for the cases with deteriorated liver function like HCC in CLD [13, 14]. There are several studies which propose alternative approaches to major hepatectomy in such cases [15, 16]. Extended sectionectomy of the liver combined with the major hepatic vein resection is often applied to such cases in order to preserve the remnant liver function and avoid postoperative liver failure. Torzilli et al. reported that more than half of their cases, which had been candidates of right hemihepatectomy, could undergo extended posterior sectionectomy instead of hemihepatectomy with R0-1 resection for HCC with thick nontumorous fibrous capsule [9]. They mentioned that the preservation of the anterior section, which is most relevant volume of the liver [17], was accomplished with the procedure. However, this procedure is complicated and difficult even in open liver resection [9]. In the setting of LLR, although the reports of laparoscopic major hepatectomy are increasing, the tumors close to the liver hilum and major hepatic veins are still considered contraindications and there are very few reports of LLR for these lesions [8]. There are only several reports even for conventional laparoscopic sectionectomies of LLR [10, 18–21]. Also, there is only one case report of left medial and right ventroanterior sectionectomy of LLR [22]. Laparoscopic extended sectionectomy of the liver combined with the major hepatic vein resection at its root is a more complicated procedure and there is virtually no report of describing technical steps of the procedure up to the present date. We reported our results and method of handling this procedure. In our knowledge, this is the first technical report for this procedure.

For the pure laparoscopic extended sectionectomy combined with the major hepatic vein resection at its root, we think steps of the procedure listed in the “method” section (Figures 4–11) are useful, taking advantages of laparoscopy-specific view from caudal direction with the good magnifies vision of hilar and dorsal areas [10, 23, 24]. The mobilization of the right liver was not usually employed in these procedures. In open right liver resection, the mobilization is often performed and the operator's left hand is put behind the liver for compressing and lifting the liver in order to control bleeding and also as a guide of transection line. However, in LLR, there is chest-abdominal wall without incision in front of the liver and not enough space for lifting the liver up; besides there is no good instrument substituting the operator's left hand. In order to obtain good view of cutting surface using the gravity and postural changes, the dissection of the retroperitoneal attachments was performed at the final step of the surgery after parenchymal transection even in extended right posterior sectionectomy of LLR in our procedures. We had previously reported and described a caudal approach to posterior sectionectomy with parenchymal transection prior to mobilization of the liver [10]. In this procedure, the patient was placed in a left lateral position, in which the cutting plane of posterior sectionectomy turns to be vertical from horizontal. The posterior section was not mobilized and was fixed to the retroperitoneum. The root of right hepatic vein was minimally dissected only to confirm the end-point of transection plane at the first step. Dissection of the inferior vena cava anterior wall behind the liver and transection of the liver with the exposure of right hepatic vein simultaneously proceeded toward the bifurcation of right hepatic vein and IVC in the caudal to cranial one-way direction. Since the remnant liver sinks down and the resected liver was fixed to the retroperitoneum, the cutting surface was well opened and the good exposure of right hepatic vein for the control of bleeding was facilitated. Also, since the right hepatic vein and cutting surface were raised vertically from IVC in this setting, the venous pressure in right hepatic vein was decreased and the blood did not pool on the cutting surface.

Pringle maneuver was applied to all cases. Although CUSA, BiClamp bipolar forceps, and irrigation monopolar electric cautery with soft-mode coagulation, beside Pringle maneuver and the control of pneumoperitoneal pressure, were used for hemostasis and dissection without bleeding, acquirements of good view for operative fields using the gravity, postural change, and laparoscopic fine magnified view from caudal direction are essential in the safe dissection for central deep part of major hepatic veins. Suturing technique of the bleeding points is also mandatory. The use of energy device on the major vessels, especially on the Glissonian pedicle, should be avoided with the use of CUSA/crushing method and Pringle maneuver at the dissection of major vessels. When a small branch was pulled out and bled on the major vessels, the bleeding should be controlled by clamping or compressing with forceps/gauze and making stitch and suturing afterword (we usually use the so-called fisherman's knot with 4-0 or 5-0 PROLENE under Pringle maneuver and temporary higher pneumoperitoneum pressure up to 14 mmHg with gradual stepwise increase). When the resection is completed, the points of small bile leakage on the Glissonian pedicle should be well examined and repaired with stitch and suturing under the laparoscopic magnified view.

We think we can acquire the benefits of laparoscopic good magnified view from caudal direction (especially for the hilar and dorsal area of the liver) using these standardized steps. They could make this complicated procedure feasible and safe. Contrary, laparoscopic approach has a weakness in overview of the operative field and is easily getting into disorientation. For example, the proper transection plane between the caudate lobe and anterior/medial sector is hard to be defined during parenchymal transection. However, in LLR, the laparoscopic view is horizontal and in the caudal to cranial direction, which is parallel to the transection plane spread from hilar plate to the roots of hepatic veins (Figure 5). After the confirmation of end point of transection (the roots of hepatic veins) in the first step of the surgery, following the horizontal plane from hilar plate to the end point along with the side-dissection of preserving major hepatic vein surface was carefully performed under laparoscopic view in our cases. We still do not have the experience of performing this procedure in the case of repeat liver resection. Since the alteration of the structures in the liver could be difficult to perform this procedure under laparoscopic setting in those repeat treatment cases, we need to do well-examined preoperative simulation using 3D-CT imaging. From our experience, 3D laparoscope was also helpful to recognize the positional relationship between structures and to obtain the good overview of the operative field. Further examination is needed.

## Figures and Tables

**Table 1:** Summary of the patients who underwent extended sectionectomy combined with major hepatic vein resection.

Number	Age, sex	Operative procedure	Disease	Tumor size (mm)	Operation time (minutes)	Bleeding (mL)	Oral intake restored	Complication	Hospital stay (days after surgery)
1	73, M	Right anterior	HCC	48	352	480	1 POD	—	21
2	70, M	Left medial	HCC	75	466	350	2 POD	—	44 due to warfarin control
3	58, M	Right anterior	HCC	70	603	750	2 POD	Pleural effusion	44
4	70, M	Right posterior	HCC	40	506	100	1 POD	—	16
5	78, M	Left medial	HCC	72	341	250	2 POD	—	8
6	79, M	Right anterior	HCC	50 (VTT)	401	250	1 POD	—	15

M: male; HCC: hepatocellular carcinoma; POD: postoperative day, VTT: venous tumor thrombus.

Case numbers are listed in chronological order in our experience.

**Table 2:** Comparison between the patients who underwent extended sectionectomy combined with major hepatic vein resection and the patients who underwent other anatomical resections.

	Extended sectionectomy (*n* = 6)	Other anatomical resections (*n* = 29)
Age	58–79 (72)	57–82 (72)
Male : female	6 : 0	17 : 12
Disease HCC : others	6 : 0	13 : 16
Tumor size (mm)	40–75 (60)	12–145 (23)^*∗*^
Operation time (minutes)	341–603 (434)	217–848 (401)^*∗*^
Intraoperative bleeding (mL)	100–750 (300)	NC–3569 (181^*∗*^
Hospital stay (days after surgery)	8–44 (18)	8–52 (15)^*∗*^
Complications (Grade II or above)	1/6	5/29^*∗*^
Mortality	0/6	0/29^*∗*^

Numbers are indicated as range (median).

NC: not countable.

^*∗*^Not significantly different from “extended sectionectomy” group.
